# No Habitat Selection during Spring Migration at a Meso-Scale Range across Mosaic Landscapes: A Case Study with the Woodcock (*Scolopax rusticola*)

**DOI:** 10.1371/journal.pone.0149790

**Published:** 2016-03-22

**Authors:** Ariñe Crespo, Marcos Rodrigues, Ibon Telletxea, Rubén Ibáñez, Felipe Díez, Joseba F. Tobar, Juan Arizaga

**Affiliations:** 1 Department of Ornithology, Aranzadi Sciences Society, Zorroagagaina 11, E20014 Donostia-S. Sebastián, Spain; 2 GEOFOREST Group, IUCA, Department of Geography and Land Management, University of Zaragoza, Pedro Cerbuna 12, E50009 Zaragoza, Spain; 3 Club de Cazadores de Becada, Avda. Schulz 8, 4 dcha, E33208 Gijón, Asturias, Spain; Università della Tuscia, ITALY

## Abstract

Success of migration in birds in part depends on habitat selection. Overall, it is still poorly known whether there is habitat selection amongst landbird migrants moving across landscapes. Europe is chiefly covered by agro-forestry mosaic landscapes, so migratory species associated to either agricultural landscapes or woodland habitats should theoretically find suitable stopover sites along migration. During migration from wintering to breeding quarters, woodcocks (*Scolopax rusticola*) tagged with PTT satellite-tracking transmitters were used to test for the hypothesis that migrants associated to agro-forest habitats have no habitat selection during migration, at a meso-scale level. Using a GIS platform we extracted at a meso-scale range habitat cover at stopover localities. Results obtained from comparisons of soil covers between points randomly selected and true stopover localities sites revealed, as expected, the species may not select for particular habitats at a meso-scale range, because the habitat (or habitats) required by the species can be found virtually everywhere on their migration route. However, those birds stopping over in places richer in cropland or mosaic habitats including both cropland and forest and with proportionally less closed forest stayed for longer than in areas with lower surfaces of cropland and mosaic and more closed forest. This suggests that areas rich in cropland or mosaic habitat were optimal.

## Introduction

Migration is highly energy-demanding, and consequently has deep implications on migratory organisms’ life-histories (e.g., [1, 2]). Bird migration is commonly divided into periods of flight interrupted by stopovers, when birds replenish their fuel reserves, rest or simply wait up to the next flight bout [3, 4]. Most of the time of migration is consumed at these stopover sites [5, 6]. Thus, understanding when, how and why birds organize their stopover strategies is crucial from ecological, evolutionary and conservation perspectives [3, 6–8].

Habitat selection during migration can have direct consequences on success of migration [6, 7], but also on other life-history aspects through carry-over effects [9–11]. Thus, some habitats will be associated to more abundant or higher-quality resources [12]. In those species using discrete habitats, such as waterbirds, selection is easy to predict, because the species’ requirements meet in the same type of landscape. In contrast, less understood is habitat selection amongst landbird migrants moving across mosaic landscapes [8]. Although in species using a broad range of habitats the resource may be continuum and difficult to predict, selection could still occur, because habitat selection process may operate at distinct levels [13, 14]. For instance, a terrestrial bird could show relatively weak requirements at a meso-scale level (length from 1 to 100 km), when deciding to land at a particular site (whether the site may provide a minimum amount of the key resources, e.g. some woodland or open habitats). However, once landed the bird may select for more specific habitats (e.g., [15]).

Overall, relatively little attention is paid to those migrants that cross apparent suitable areas throughout which they would potentially stop over everywhere. Some previous works state that landbird migrants crossing Europe may not have a strong need to select a site to land (e.g. [16]), especially if they may not depend on patchily distributed habitats, such as wetlands. Most Europe is chiefly covered by an agro-forestry mosaic or mosaic of pastures, croplands and semi-natural forest patches [17]. In this scenario, several migratory species associated to either woodland habitats or agricultural landscapes should theoretically find suitable stopover sites along most or even the whole route of migration from their breeding quarters in northern Europe up to southern Europe [18]. Hypothetically, one may then expect no habitat selection, at least at a meso-scale level. Although repeatedly suggested, this is a question poorly documented and to the best of our knowledge, an untested hypothesis.

During the winters of 2006 to 2012, 20 Eurasian woodcocks (*Scolopax rusticola*) wintering in northern Iberia (southern Europe) were tagged with PTT satellite-tracking transmitters, allowing us to obtain a good data set of the stopover sites used along their flyway to their breeding areas in Russia [19]. The aim of the present article is to describe the habitat use of this terrestrial, game bird throughout Europe, and to test the hypothesis that, provided that most of Europe is covered by an agro-forest mosaic constituting woodcocks’ preferred habitat, there is no meso-scale habitat selection during migration.

## Materials and Methods

### Ethics statement

Authorizations to capture, ring and mark woodcocks with PTTs were provided by Regional Councils or Governments of: Asturias, Gipuzkoa, Cataluña, Baleares, Castilla y León, Andalucía, Galicia, Bizkaia and Navarra.

### Avian model

The Eurasian woodcock is a widespread wader occurring over much of the Palaearctic [20]. It mainly breeds in boreal and temperate forested habitats, from Portugal to the East of Asia but also in some sub-tropical Atlantic archipelagos in Macaronesia [21, 22]. Northern woodcocks are migratory and overwinter mainly in southwestern Europe [20, 23]. The species commonly depends on grasslands or other similar habitat types (including croplands) to feed, but during the day it moves to woodland habitats [24, 25].

The migration of woodcocks wintering in Spain is well described in Arizaga et al. [19]. In spring, these birds show a rather direct route to their breeding areas in (mainly) Russia (mean distance: 6522 km, SE = 434 km, *n* = 12). The mean axis of these spring routes cross all Europe, passing through France, Germany, Poland, with then some birds taking a more northern flyway to arrive in Finland and Western Russia, whilst others take a more southern flyway passing through Ukraine and Southern Russia, up to Central Siberia [19].

### Sampling methods

From 2006 to 2013, 20 woodcocks wintering in Spain were tagged with PTTs in several regions. From them, nine birds were considered for the analyses of this study (S1 Table). Woodcocks were captured when foraging in meadows at night, using a 12 V-100 W lamp attached to a helmet [24, 26, 27]. Once a bird was detected the observer (ringer) approached to it very cautiously, in straight line. Woodcocks were captured with a big circular net attached to a pole. We captured no more than two birds per night, and the second bird was not caught up to the first one was tagged and released. Overall, all the process did not take us >10 min. Occasionally, some birds were found to lose some small body cover feathers (<5 feathers), but never the flight (wing or tail) feathers. The PTT was attached to the bird with a home-made back-pack nylon harness. Woodcocks’ weight when caught ranged from 320 to 385 g (mean ± SE: 348 ± 6 g), and hence the weight of the PTT did not exceed the 3.0% (mean: 2.8%) of woodcocks’ body mass. This value is below the upper recommended weight that a device like a PTT on a bird can have [28]. Although we did not test for the possible side effect of the PTT [29], our own experience suggests that these effects might be negligible, as tagged birds were shown to have an apparently normal behaviour (e.g. home range area and mean departure date similar to what it is reported in other works).

Most woodcocks were tagged at the end of the winter period, once the hunting season had finished at the region where the birds were marked (range: 10 Feb.–15 Mar.; S1 Table). This was done with the aim of avoiding that the birds may be shot before the spring migration [30].

### Characteristics of the PTTs

The PTTs were produced by MTI, Columbia (USA). PTTs dimensions were: 38×17×12 mm, 9.5 g. All the PTTs were equipped with a battery charged by a solar array. Most PTTs (*n* = 6) were programmed to charge energy during a period of 48 h, followed by a 10-h emission period (i.e., a 48:10 cycle). Alternative duty cycles were also proved (55:08; for details see S1 Table). Emission periods were planned to coincide with the day, although we also obtained data at night. Accordingly, we were forced most PTTs to have 2-days “gaps” each 10-h of emission. If the satellite did not pass over the PTT during that 10-h period (or the signal was too low to be detected), then the temporal gap extended to a minimum of 106 h (i.e., 4.4 days).

Data provided by the satellites and used here were: (1) platform (bird) identification, (2) date and hour when the signal was obtained (actual position of a bird), (3) accuracy of the position and (4) position (latitude, longitude). Argos provides a location accuracy of Z, B, A, 0 (>1500 m), 1 (500–1500 m), 2 (250–500 m) and 3 (<250 m). The Z category just indicates that the satellite was able to detect the PTT but unable to assess a geographic position (no accuracy). Categories A and B have no accuracy estimation, but it can be of even >1° (i.e. >100 km) both in latitude and longitude.

### PTTs data selection

Data considered in this study were only relative to the 0 to 3 categories (i.e., good quality data; categories Z, B and A were excluded). At their wintering sites, distance from a position to the next did not exceed 0.3° (ca. ~30 km as determined by Argos). To be conservative, we considered that subsequent points (positions) situated at a distance <0.4° belonged to a same site. When more than one location was obtained for a particular site, we calculated the median to obtain a more realistic position of the bird at that site. Thus, during migration period, we determined a stopover site as the median position of a number (≥ 2) of subsequent points situated at a distance <0.4°. However, some of such “apparent” stopovers concerned to staying periods <24 h, and in some cases it would be difficult to confirm whether the bird was really stationary or on migration. Therefore, a site was considered to be a stopover site if the bird was there ≥2 days (Table 1).

**Table 1:** Number of locations of individual (ID) woodcocks during the spring migration. Of 20 woodcocks tagged in this project overall, we only considered for the analyses of this study those birds for which one or more stopovers of >24 h were detected (*n* = 9 woodcocks).

ID	1 position	Stopovers (<24 h)	Stopovers (>24 h)
04	1	0	1
05	4	0	1
09	2	0	3
10	3	3	2
11	6	8	6
16	3	5	4
17	0	2	2
19	6	3	4
20	3	1	3

### Habitat data

We used the GlobCover 2009 (GC) cartographic project to analyze the habitat use at the stopover localities. GC provides a global coverage with a resolution of 300 m, and identifies 23 soil covers (for details see http://due.esrin.esa.int/globcover/).

We were unable to obtain an accurate estimation of the home range area of our birds at their stopover sites, since most of them provided an insufficient number of points (<30) to obtain an acceptable assessment [31, 32]. Thus, we considered a fixed home range area within a radius of ±0.2° (equivalent to ~20 km) around the median position of each individual bird at each stopover site [19]. This comprises a surface similar to the greatest home range area detected during the winter period in Iberia [19]. Given this, we calculated the percentage of each type of soil cover for the sites where the birds were detected to stop over.

**Fig 1:**
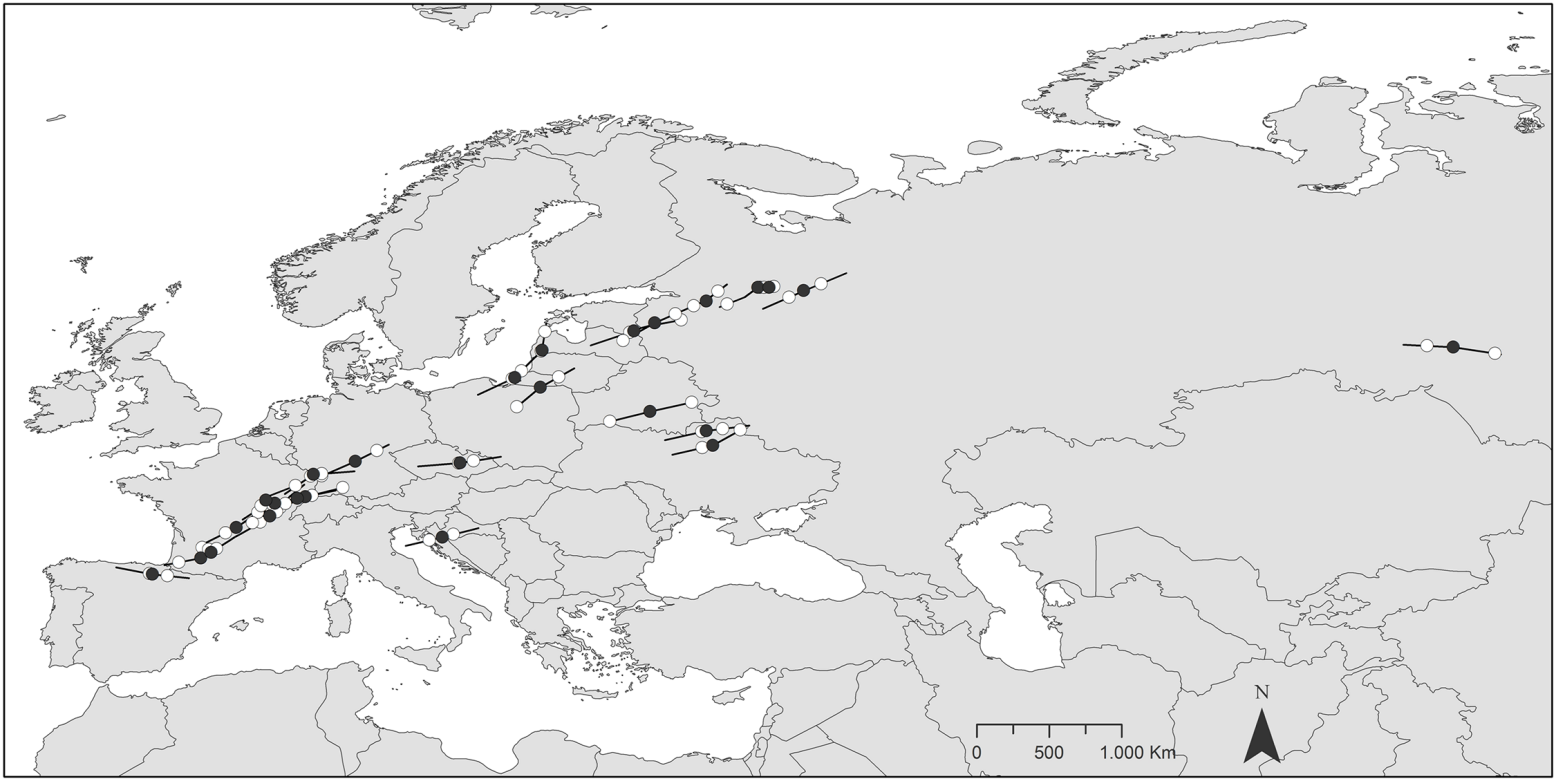
Example (replication 1) of the location of the stopover localities (closed dots) and the two randomly selected points (open dots) along the route of migration of nine woodcocks in spring.

In parallel to these stopover sites, we randomly selected two additional points situated within a transect of ≤184 km from each stopover site. The length of the transect was determined since the speed of migration was estimated to be 184 km/day [19]. Random points were situated along the route (linear segments connecting subsequent stopover localities) of migration of each bird; they were selected with ArcMap 10.2 random points selection tool (Fig 1). This procedure was replicated four times for each bird and the four replications were decided because within a distance of 184 km we can only find four non-overlapping “areas” with a radius of 0.2°. Migration routes were delineated as the sum of the linear segments which linked subsequent stopover localities from winter to breeding quarters [33].

To obtain un-correlated proxies on habitat type, we conducted a Principal Component Analysis (PCA) on the proportion of soil covers (here also understood as habitat type). Overall, we obtained five components (PC) which in total explained a 92.6% of the variance (Table 2).

**Table 2:** Habitats identified in the spring stopover localities (and random points) of woodcocks migrating to their breeding sites in Russia and correlation coefficients with the most important components (PC) from a PCA on the whole habitat types. Values in bold indicate a significant weight of that variable (habitat) on the PC. This PCA was carried out considering the four replicates.

Code	Code meaning	PC1	PC2	PC3	PC4	PC5
CRO1	Post-flooding or irrigated croplands (or aquatic).	+0.001	+0.001	+0.001	+0.001	-0.002
CRO2	Rainfed cropland.	**+0.428**	**+0.584**	+0.019	-0.029	**-0.593**
MOS1	Mosaic cropland (50–70%) + vegetation (grassland/shrubland/forest) (20–50%).	+0.242	+0.265	**+0.383**	**+0.477**	**+0.531**
MOS2	Mosaic cropland (20–50%) + vegetation (grassland/shrubland/forest) (50–70%).	+0.125	+0.063	**-0.365**	-0.067	+0.373
BFO2	Closed (>40%) broadleaved deciduous forest (>5m).	+0.053	**-0.583**	-0.077	**+0.615**	-0.364
NFO1	Closed (>40%) coniferous (needleleaved) forest (>5m).	+0.011	-0.010	+0.112	+0.005	+0.166
NFO2	Open (15–40%) coniferous forest (>5m).	-0.031	-0.135	+0.219	**-0.378**	-0.050
MFO1	Closed to open (>15%) mixed broad-leaved and coniferous forest (>5m).	-0.035	-0.284	**+0.439**	**-0.400**	+0.037
MOS3	Mosaic forest or shrubland (50–70%) + grassland (20–50%).	-0.010	-0.037	+0.020	-0.024	-0.043
MOS4	Mosaic forest or shrubland (20–50%) + grassland (50–70%).	+0.076	-0.068	-0.625	-0.157	+0.093
SHRU	Closed to open (>15%) shrubland (<5m).	+0.001	+0.001	+0.005	+0.004	-0.001
GRAS	Closed to open (>15%) herbaceous vegetation (grassland, savannas or lichens/mosses).	+0.024	-0.013	-0.237	-0.094	+0.154
SVEG	Sparse (<15%) vegetation.	-0.007	-0.040	+0.029	+0.009	-0.072
FVE2	Closed to open (>15%) wood- or grassland regularly flooded or waterlogged soil.	-0.033	-0.106	+0.107	-0.153	-0.123
URBA	Artificial surfaces (urban areas >50%).	+0.011	+0.007	-0.001	+0.023	-0.011
BARE	Bare area.	+0.000	+0.000	+0.000	+0.000	+0.000
WATE	Water body.	**-0.854**	+0.356	-0.032	+0.169	-0.094
Eigenvalue		0.072	0.059	0.018	0.013	0.011
Variance (%)		39.13	29.94	10.79	6.79	5.80

### Data analyses

To test whether soil covers at stopover sites differed from those found at points randomly selected along the route of migration we conducted Generalized Linear Mixed Models with the type of “stopover” as a binary object variable (stopover versus random points) and the (logit) link function with binomial errors distribution. The PC were included as linear predictor variables and the individual ID (i.e. PTT Platform) as a random factor. We obtained two random points for each stopover site, and this was done 4 times (i.e. we obtained 4 replicates, with 2 random points within each replication, for each stopover site). Then, all the possible additive models were built using the “dredge” function in R for each set of replicates [34]. Models were ranked in relation to their small-sample size Akaike values (AICc); those models which differed in less than 2 ΔAICc were considered to fit the data equally well [35]. Model averaging [35] was carried out across the models within the subset of models with an ΔAICc<2 in relation to the top one. After this, we built a final, global averaged model, which resulted from averaging all the four previously averaged models. Averaged final and per each replicate models β-parameter estimates, their standard error and the associated *P*-values were obtained. The effect of a variable was non-significant for *P*-values >0.05. To minimize spatial autocorrelation effects, original data were randomly sub-sampled until Moran index showed no significant correlation (i.e. *P* values >0.05) in their model residual values [36]. This procedure was carried out separately for each replication.

Given that the proportion of each soil cover type may be influenced by the buffer size, we conducted a second analytical approach contributing to test randomness in habitat characterization. Taking into account the minimum accuracy in stopover location provided by the PTTs (1500 m), we calculated a number of buffers of different size at intervals of 1500 m (overall, 14 buffers ranging from 1500 to 21000 m). As before, we calculated within each buffer the percentage of each soil cover type, and from here ran a new PCA and the corresponding Generalized Linear Mixed Models. This analysis was carried out for each buffer, with each individual ID included as a random factor. We only used in this case the stopover and random points considered in replication 1 (Fig 1). Models were compared in terms of their AICc value [35].

To analyze whether the stopover duration and its geographic location were associated with soil covers we conducted pair-wise Spearmann linear correlations between the stopover duration and the geographic location (longitude) and the PCs obtained for the 0.2° buffer. Since this is a case of multiple testing we applied a Bonferroni correction for the significance level (up to *P* = 0.005, as we ran ten correlations).

All analyses were run with R [37], and the “lme4” [38], “MuMIn” [34] and “psych” [39] packages.

## Results

**Fig 2:**
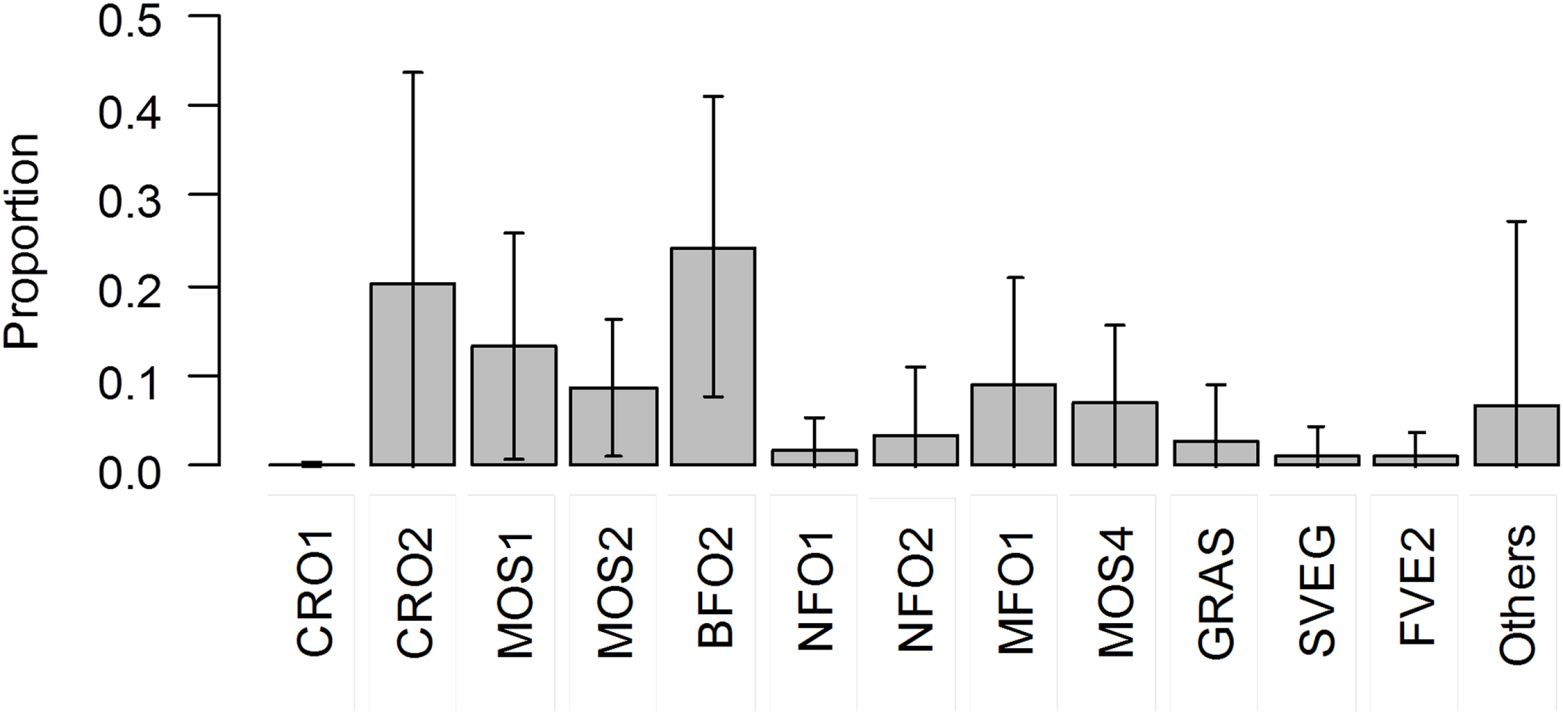
Mean (±SD) relative area comprised by each habitat at the stopover localities of woodcocks in their spring route to their breeding sites in Russia. Habitat abbreviations as in Table 2.

We analysed data on 25 stopover sites in total, relative to nine woodcocks. Overall, woodcocks were observed to stop over in places with ca. 25% of closed broadleaved forest (either deciduous or evergreen), 20% of cropland, and 30% of mosaic habitats most of which included distinct types of cropland together with other forest, shrub or grass patches (Fig 2).

The proportion of each soil cover type (summarized as PC1 to PC5) was not useful to separate the stopover localities from points randomly selected along the route of migration (Table 3), indicating that woodcocks did not seem to select any given soil cover to stop over.

**Table 3:** β-parameter estimates, their standard error and the associated *P*-values obtained from a final averaged model derived from the four averaged models used to test for the effect of soil cover types (summarized by means of a PCA) on whether a site was a true stopover site or a random point across woodcocks migration routes.

Variables	β	SE (β)	*P*
**Intercept**	-1.543	0.471	0.001
**PC1**	+0.581	1.490	0.697
**PC2**	-0.001	0.175	0.990
**PC3**	-4.934	3.297	0.140
**PC4**	+6.251	5.039	0.689
**PC5**	-3.895	3.752	0.379

Considering the second analytical approach using home range buffers of several sizes, we observed that almost all the models fitted to the data equally well (Table 4), indicating that the size of the buffer did not have a statistically relevant effect to predict our capacity to separate true stopover sites from those randomly selected in relation to the PCs that summarized the proportion of each soil cover type. Thus, the soil cover type was likely to be rather homogeneous around each stopover site, and did not depend on the area considered around the median geographic location of the home range area. A detailed look at the averaged β-parameters (±SE, *P*) showed that none of the PCs had any predicting capacity (Intercept: -0.713 ± 0.251, *P* = 0.005; PC1: -0.024 ± 0.957, *P* = 0.980; PC2: -0.544 ± 1.282, *P* = 0.677; PC3: -0.060 ± 1.845, *P* = 0.974; PC4: +2.348 ± 2.233, *P* = 0.301; PC5: -2.129 ± 2.265; *P* = 0.355).

**Table 4:** Models ranked in relation to their small-sample size Akaike values (AICc) used to test for the effect of soil covers (assessed with PCs 1 to 5) on the differentiation of stopover localities from randomly selected points.

Buffer size (km)	AICc	ΔAICc	Deviance
6.0	106.62	0.00	92.62
10.5	106.62	0.00	92.62
9.0	106.67	0.05	92.67
7.5	106.79	0.16	92.79
15.0	106.87	0.25	92.87
18.0	106.95	0.33	92.95
12.0	106.97	0.35	92.97
13.5	106.98	0.36	92.98
16.5	107.03	0.41	93.03
19.5	107.21	0.58	93.21
21.0	107.24	0.62	93.24
4.5	107.70	1.08	93.70
3.0	108.48	1.85	94.48
1.5	109.11	2.48	95.11

Finally, we observed an almost-significant correlation between the stopover duration and the PC2 and PC3, indicating that woodcocks tended to lengthen their stopover when they landed in places proportionally richer in cropland or mosaic habitats including cropland and forest and with proportionally less closed forest (Table 5). Moreover, the PC1 was observed to be negatively correlated to longitude, indicating that stopover sites situated to the east had proportionally less cropland, and more closed to open mixed forest areas (Table 5). This shows that birds passing through eastern Europe and Russia found a higher proportion of forest in detriment of cropland habitats.

**Table 5:** Spearmann (*rho*) coeficients of correlation used to test for the existence of significant correlation (if *P*<0.05) between the stopover duration and the location (longitude) of each stopover site and the soil covers (assessed with the PCs obtained from a PCA on all the soil covers in a buffer of 0.2° radius).

	Stopover duration		Location (longitude)	
	*rho*	*P*	*rho*	*P*
**PC1**	-0.240	0.25	-0.569	<0.001
**PC2**	+0.467	0.02	+0.163	0.44
**PC3**	-0.452	0.02	+0.417	0.04
**PC4**	-0.067	0.75	+0.293	0.16
**PC5**	+0.127	0.55	-0.016	0.94

## Discussion

Although we tagged 20 woodcocks, only half of these birds provided data for the analyses and even in these cases these data were relatively scarce. The reason for this low data retrieving is technical. Woodcocks are nocturnal migrants and during the day they remain in shadowed habitats (woodlands) that hamper battery charge. This became particularly clear for the first PTTs, which were shown to have batteries with lower charging capacity (J. F. Tobar, pers. obs.).

Woodcocks moving across Europe in their route to their breeding areas did not stop over in places with a different soil cover type (i.e. habitats) than points randomly selected. This result suggests that habitats seemed to be rather uniform through the whole migration route, indicating that woodcocks move across a relatively uniform landscape chiefly composed by an agro-forest mosaic (although with a higher proportion of forest in the east and a higher proportion of cropland habitat in western Europe). Since this mosaic including forest, cropland or natural meadows is used by woodcocks [25, 26, 40], they might take clear advantages from this scenario, which would allow them (1) to stop over in familiar habitats, so to minimize those risks associated to landing in unknown habitats, and (2) to have no need to deal with physiological or behavioral adaptations associated with the exploitation of alternative resources [8]. This habitat-related uniformity would also allow woodcocks to virtually ignore potential risks associated to emergency landing in case of adverse weather [41].

If the habitat has a negligible role on woodcocks landing decisions, then other factors would be much more important, such as the end of the night (if it is true that woodcocks are nocturnal migrants) [20], weather and the current physiological condition [42, 43]. This is something to be solved in future studies. Woodcocks landing in zones with more broad-leaved forest and grasslands had longer stopovers, hence the habitat may influence stopover duration. Although stopover duration can vary in relation to many causes, including predator densities, weather, food availability, distance to next site or destination, etc. [4], it is commonly seen that a long stopover is associated to good conditions for refuelling, normally in terms of food availability [44, 45]. Such conditions would allow high rates of fuel accumulation [46–48]. Thus, although at a meso-scale level we had no evidence supporting habitat selection during migration, those birds stopping over where it might be better, more optimal habitat, stayed for longer. Also, stopover duration may be related to the previous flight distance: a bird might need more time to refuel after a long flight than after a short flight. Woodcocks could hence have a hierarchical stopover strategy: they could first land in apparently good zones, where they would look for a mosaic of forests and meadows or cropland; second, once on ground, they would look for good foraging sites, which could be associated to particular forests or meadows, allowing them to gain more fuel.

In conclusion, this is the first study testing for habitat selection at a meso-scale range during migration of a landbird, agro-forest-associated species moving through Europe in spring. From a conservation standpoint, our results may indicate that woodcocks wintering in Europe may potentially use any site to stop over. Therefore, at least at meso-scale level, we would not detect species-specific habitat needs/concerns. However, this result does not mean that the species may select for particular habitats at smaller spatial scales. Noteworthy, the woodcock has been reported to show habitat selection during wintering or breeding season [24, 25]. Thus, another point would be that focusing on micro-scale aspects, such as food abundance at given crops/fields [24], which might influence stopover decisions or performance [48, 49].

## Supporting Information

S1 Table Tagging area, characteristics of the PTTs, age (juvenile or adult birds), capture body mass and date, last signal, time between the capture date and the last signal, and number of high-quality (0–3) points provided by the nine woodcocks used for this work. (PDF)
