# Supplementary material for: No Habitat Selection during Spring Migration at a Meso-Scale Range across Mosaic Landscapes: A Case Study with the Woodcock (Scolopax rusticola)
Source: PLoS One. 2016 Mar 22;11(3):e0149790. doi: 10.1371/journal.pone.0149790 (PMC4803235; doi:10.1371/journal.pone.0149790)
Supplement: S1 Table — (PDF) [file pone.0149790.s001.pdf]

S1 Table. Tagging area, characteristics of the PTTs, age (juvenile or adult birds), capture body mass and date, last signal, time between the capture date and the last signal, and number of high-quality (0-3) points provided by the nine Woodcocks used for this work.

ID	Coor.	Duty cycle	Age	Body mass (g)	Starting date	Last signal	Elapsed time (days)	No. points (0-3)
04	42º54’N 02º06’W	48:10	Ad	360	02/03/2007	12/08/2008	529	47
05	43º00’N 02º40’W	55:08	Jv	385	08/03/2008	08/08/2008	153	23
09	42º54’N 02º07’W	55:08	Ad	325	10/02/2010	25/05/2010	104	45
10	43º20’N 04º04’W	55:08	Ad	350	19/02/2010	13/11/2010	267	57
11	43º04’N 02º41’W	48:10	Jv	350	15/03/2010	06/09/2010	175	154
16	43º07’N 01º40’W	48:10	Ad	340	10/02/2012	16/10/2012	249	441
17	43º22’N 02º40’W	48:10	Ad	340	18/02/2013	11/09/2013	205	119
19	43º07’N 02º23’W	48:10	Ad	320	20/02/2013	24/08/2013	185	89
20	43º43’N 07º56’W	48:10	Ad	370	01/03/2013	11/09/2013	194	117
